# Why Do I Love Her

**Published:** 1892-04

**Authors:** 


### WHY DO I LOVE HER ?

Tell why I love her ? Tell me why,
Turning from murky town and pushing men,
You love the woodlawn path, the placid sky.
I'll answer then.

Why do I love her ? Analyze
Where in the violets the perfume is,
Where in the music's strain the tears arise.
Can you do this ?

Tell why I love her ? Yes, when you
Reveal the secrets which in snowdrops lie,
Or strain the beauty from the drops of dew.
Then I'll tell why.

Why do I love her ? First make clear
Whence steals through minster aisles the restful spell
That fills with mystic sense the atmosphere.
I then will tell.—Ex.

